# More than just a gel: the extracellular matrixome of *Pseudomonas aeruginosa*

**DOI:** 10.3389/fmolb.2023.1307857

**Published:** 2023-11-13

**Authors:** Rahan Rudland Nazeer, Meng Wang, Martin Welch

**Affiliations:** Department of Biochemistry, University of Cambridge, Cambridge, United Kingdom

**Keywords:** *Pseudomonas aeruginosa*, biofilm, extracellular polysaccharide, phenazine, quorum sensing, extracellular DNA, membrane vesicles, interactome

## Abstract

Armed with an arsenal of protein secretion systems, antibiotic efflux pumps, and the occasional proclivity for explosive self-destruction, *Pseudomonas aeruginosa* has become a model for the study of bacterial pathogenesis and biofilm formation. There is accruing evidence to suggest that the biofilm matrix—the bioglue that holds the structure together—acts not only in a structural capacity, but is also a molecular “net” whose function is to capture and retain certain secreted products (including proteins and small molecules). In this perspective, we argue that the biofilm matrixome is a distinct extracellular compartment, and one that is differentiated from the bulk secretome. Some of the points we raise are deliberately speculative, but are becoming increasingly accessible to experimental investigation.

## 1 Introduction

*Pseudomonas aeruginosa* (PA) is a Gram-negative organism characterised by an exceptionally diverse metabolism and a predilection for colonising anthropic niches (Brinkman et al., 2021). With a genome comprising over 5,500 open-reading frames, the organism displays remarkable phenotypic plasticity, which, at least partially, accounts for its ubiquity in the built environment. It is also an opportunistic human pathogen, responsible for high morbidity and mortality, especially in individuals who are predisposed towards infection due to co-morbidities such as cystic fibrosis or neutropenic cancer (Schmidt et al., 1996; Stover et al., 2000).

Much is made of PA’s environmental responsiveness, especially with respect to its complex network of inter-connected two-component signalling systems (TCS), and its agnosticism where electron donors and acceptors are concerned (Francis et al., 2018; Brinkman et al., 2021). PA can readily reconfigure its physiology to gain a fitness advantage over neighbouring microbes, and to deal with existential threats such as the immune system and antibiotics (Rossi et al., 2021; Rozner et al., 2022). It also constantly titrates the physicochemical features of its environment, adapting its physiology accordingly. A less well-appreciated facet of PA biology is that it also actively modifies the environment to suit its own needs. It does so through the use of secreted products, and with its plethora of secretion systems, PA can be considered a “professional secretor”.

Traditionally, bacteria have been considered to be the archetypal single-celled organisms. However, over the last few decades it has become increasingly clear that many species of bacteria, including PA, have a thriving social life. They communicate with one another (although not always in the spirit of cooperation) and often live in conglomerates known as biofilms (Costerton et al., 1999). Moreover, it is clear that a good deal of biochemical activity occurs *outside* of- and *in-between* single cells. Nutritional cross-feeding, exoelectrogenesis, protein secretion, and quorum sensing are just a few examples of the kind of mechanisms mediating this “shared goods and services” economy. Indeed, relative to the size of the individual cell, PA’s secretions have an impressive geographical reach; the microbial bailiwick extends well beyond the boundaries of the cell envelope.

## 2 Anatomy of the extracellular matrix

Many strains of PA exhibit a propensity to form biofilms, sometimes incorporating other species. These structures are essentially aggregates of cells encased in a self-produced matrix of exopolymers. Although they vary in form and appearance, biofilms represent a distinct physiological state that bookends the lifestyle spectrum, with individual planktonic cells on the opposing flank. Importantly, biofilms are associated with chronic infections, partly because the extracellular matrix confers elevated antibiotic tolerance, immune evasion and recalcitrance to ciliary clearance (Mishra et al., 2012; Ciofu and Tolker-Nielsen, 2019; Jennings et al., 2021; Hall-Stoodley and McCoy, 2022).

PA is a well-known respiratory pathogen. Crudely, successful colonisation of the airways and establishment of a chronic infection requires two things from PA. The first is concerted secretion of exoenzymes to raze the host tissue matrix (Golovkine et al., 2014; Flynn et al., 2017; Cigana et al., 2021). The second is secretion of exopolymeric substances to “glue” the PA in place (Chew et al., 2018). This “terraforming” of the lung tissue no doubt involves additional mechanisms (exclusion of competitors, and so on) but for our purposes, this basic description will do.

The biofilm matrix was originally described as an amorphous, inert slime, coating both the cells and any nearby surface. Hardly surprisingly, the resulting sessile communities manifest different transcriptomic and proteomic profiles compared with their planktonic counterparts (Whiteley et al., 2001; Mikkelsen et al., 2007; Patell et al., 2010; Erdmann et al., 2019; Thöming et al., 2020). Relevant to the current perspective, it is also increasingly apparent that biofilm-associated cells secrete an altered spectrum of proteins into the extrcellular milieu (Passmore et al., 2015). However, to access to the surrounding medium, secreted products need to first pass through the biofilm matrix, raising the question of whether some of these secreted proteins are trapped to generate a distinct matrix-associated proteome (the “matrixome”) that is different from the “true secretome”. Investigating this has not been trivial, and several teams have been involved in trying to separate matrix-embedded cells from “everything else” in the biofilm (Toyofuku et al., 2012; Couto et al., 2015). Here, we argue that the biofilm matrix does indeed comprise a distinct extracellular compartment, and one that is compositionally different from the bulk secretome that bathes it.

Collecting the biofilm-derived secretome has been challenging enough, but the task of segregating embedded biofilm cells from their adherent, sticky matrix is proving even more so. Nevertheless, some progress has been made. Studies carried out using “colony biofilms” on agar surfaces have indicated that, like the true secretome, the matrixome contains a wealth of degradative enzymes, such as peptidases, nucleases, peroxidases/catalases, as well as large fibrillar adhesins such as CdrA, and protein-laden membrane vesicles (Toyofuku et al., 2012; Couto et al., 2015). The presence of such matrix-associated proteins raises interesting questions as to whether they are captured “by accident” due to some coincidental physico-chemical property, or whether they have evolved to be active participants in extracellular matrix function. However, there has been relatively little detailed biochemical follow-up on this, and currently, no studies have been reported that examine the matrixome of biofilms grown in liquid media (i.e., conditions that are more faithfully reflect biofilm formation in infection scenarios).

One notable exception to the lack of detailed follow-on investigation relates to studies carried out by the Parsek and Howell groups, who have made substantial contributions to our understanding of the interface between the exopolysaccharide and protein components of the biofilm matrix. Through their elegant work, we know that a cyclic-di-GMP-regulated protein abundant in the biofilm matrix, CdrA, is a binding partner for both Psl and Pel exopolysaccharides, and that Pel also binds to extracellular DNA (eDNA; see below) (Borlee et al., 2010; Jennings et al., 2015; Reichhardt et al., 2020). CdrA’s interaction with its polysaccharide binding partners stabilises it against degradation and seems to be important in cell aggregation, which confers a degree of antibiotic tolerance in some experimental regimes (Reichhardt et al., 2018; Melia et al., 2021). Overall, we build up a picture of CdrA interacting with the extracellular polysaccharides, thereby physically cross-linking the matrix. But there may be more to the story than this.

**FIGURE 1:**
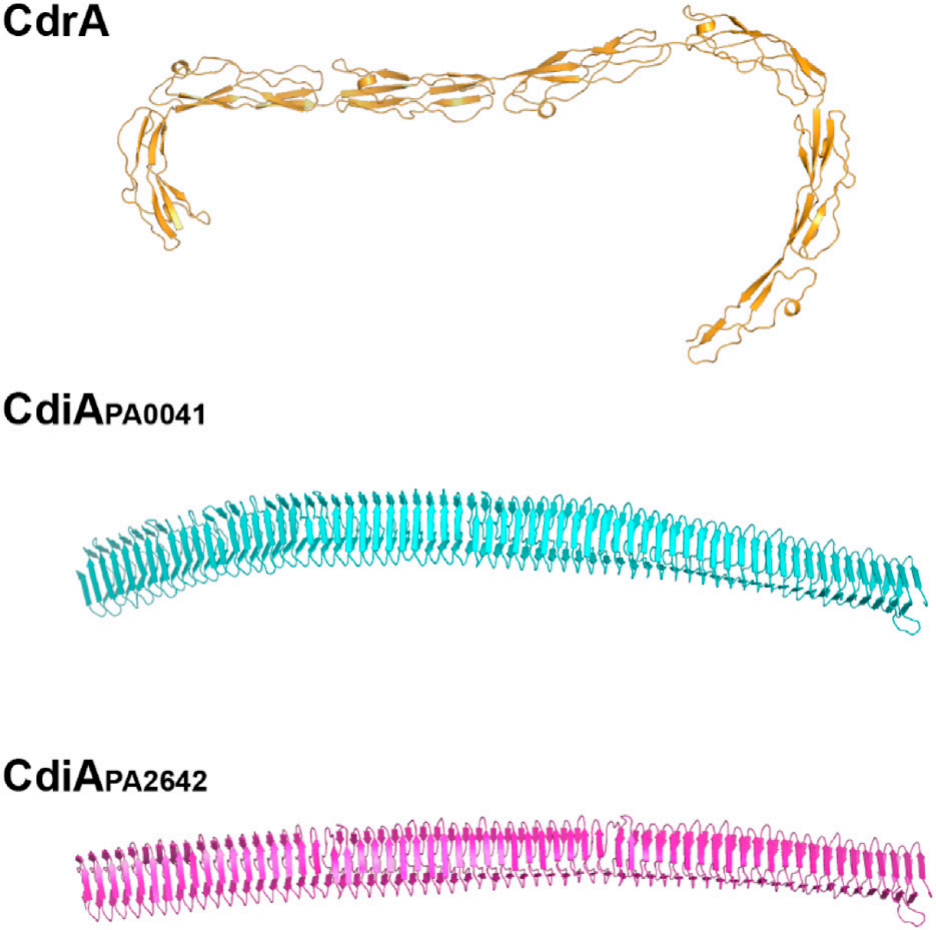
β-strand-rich extracellular domains of CdrA, CdiA_PA0041_, and CdiA_PA2642_. We speculate that these “fibrillar” proteins may share common functions in both contact-dependent inhibition and adhesion to the biofilm matrix.

CdrA is also found associated with the surface of cells. The extracellular portion of CdrA is predicted to comprise multiple repeats of a β-strand rich domain, which self-assemble to form a rigid, matchstick-like projection from the cell surface (Melia et al., 2021). Several other PA proteins contain similar β-rich repeat motifs, including the cell contact-dependent inhibition proteins, CdiA_PA0041_ and CdiA_PA2642_, and the secreted protease, LepA (Figure 1). Anthropocentrically, such arrangements evoke a possible protective function [as has been suggested for CdiA (Mercy et al., 2016)], and we speculate whether CdrA may also have a role in contact-dependent inhibition (in addition to its role as a molecular “spar” that cross-links matrix Pel/Psl, and possibly also physically attaches embedded cells to the matrix).

Meanwhile, in our own work, we have found other cell-associated appendages to be enriched in the biofilm matrix. For example, phage-related R-type pyocins are abundant in the matrixome (Nazeer and Welch, *manuscript in preparation*). These proteins, which resemble the contractile tails of P2 phages and can bind surface receptors of neighbouring cells before depolarizing their cytosols, are known to be used for close-quarters inter-strain and inter-species combat (Jacob, 1954; Michel-Briand and Baysse, 2002; Olubukola et al., 2019). Interestingly, and like other secreted PA proteins, pyocin proteins manifest as “charge trains” following 2D gel electrophoresis; a feature that is usually indicative of post-translational modification (Nouwens et al., 2002; Passmore et al., 2015; Forrest and Welch, 2020). Detailed analyses of these charge trains indicates that the individual protein spots that comprise them are indeed increasingly modified as the charge train is traversed, and that these modifications are located on surface-exposed residues (Forrest and Welch, *unpublished data*). Little is known about this modification process, or its function, but given the exposure of secreted proteins to the outside world, it is possible that these post-translational modifications may have evolved to facilitate immune evasion or to protect against proteolysis.

A complete picture of the biofilm matrix must include not just those chemical species which have been actively exported through secretion systems or dedicated transporters, but also the large contingent of molecules which have arrived there by means of less well understood pathways, such as membrane vesiculation (Schooling and Beveridge, 2006; Bonnington and Kuehn, 2014) or explosive cell lysis (Turnbull et al., 2016). One such molecule, and a particularly important constituent of the matrix, is DNA, and its mere presence prompts intrigue.

## 3 eDNA and moonlighting proteins

In the early 2,000s, researchers serendipitously discovered that the extracellular biofilm matrix contains an abundance of DNA. Those researchers challenged their biofilms with DNaseI, finding that it inhibited biofilm formation and up to a point, could even dissolve pre-formed biofilms (Whitchurch et al., 2002). Indeed, inhaled DNase is now a well-established treatment for cystic fibrosis-associated airway infections, and has even recently been shown to influence ecological interactions between PA and the other microbial denizens in the CF airways (Ho et al., 2023). The presence of DNA outside of the cell raises two key questions: how does it get there, and what does it do once it is there?

Around 15 years after its discovery, a mechanism for the genesis of extracellular DNA (eDNA) was proposed. The activity of a latent prophage endolysin encoded within a pyocin gene cluster (see above), was postulated to drive explosive cell lysis, thereby releasing DNA and also generating membrane vesicles (MVs) (Turnbull et al., 2016). This mode of “secretion,” based on altruistic suicide, is certainly an unconventional one. Rather than a quorum of cells modestly pumping out DNA in concert, a discreet sub-population sacrifice themselves for the benefit of the community.

Explosive lysis may represent a cellular process that has been repurposed as a means of liberating public goods. An important corollary of this is that liberated eDNA is often accompanied by passenger proteins (Turnbull et al., 2016). Consistent with the notion of passenger protein secretion, the matrixome contains an abundance of proteins with DNA binding capabilities (Toyofuku et al., 2012; Couto et al., 2015). This raises the possibility that such passengers may have evolved “moonlighting” roles in the extracellular compartment. Whether the enrichment of these proteins in the matrixome is simply indicative of their greater stability when bound to DNA, or whether it is the signature of extracellular function, remains to be determined. An intriguing possibility is that transcription factors and histone-like proteins often oligomerise on DNA, potentially leading to increased stabilization of the DNA against degradation, cross-linkage of different DNA strands, or induced strain through compaction of the DNA structure.

## 4 Phenazines and eDNA

Cells buried deep within the anoxic microenvironments of a biofilm experience electron acceptor limitation. To prevent metabolism from grinding to a halt, PA employs diffusible phenazines as mobile electron carriers. Their role is to pick up electrons derived from cellular metabolism and carry these to the nearest suitable oxidizing agent (usually oxygen, near the surface layer of the biofilm). The oxidized phenazines then return to the centre of the biofilm and repeat the process. Phenazines are so important to PA that it encodes two differentially-regulated phenazine biosynthetic clusters (Recinos et al., 2012). Minor biosynthetic modifications enable the midpoint potential of these redox species to be tweaked, such that the producer cell can even dictate which stratum of a biofilm the phenazine can collect electrons from, and with which other species it can interact (Schiessl et al., 2019; Saunders et al., 2020). The elephant-in-the-room in the above discussion is the question of why don’t oxidized phenazines just diffuse away from the biofilm? Why should they diffuse back into the core of the structure? A possible explanation is that phenazines also bind to eDNA, and that this helps to trap them in the biofilm matrix. Furthermore, and taking advantage of the “electron wire” property of DNA (related to its base stacking), phenazines can also deposit and pick up electrons from eDNA (Saunders et al., 2020). The interaction of phenazine species with DNA invokes a matrix electrical infrastructure in which DNA is threaded across the microbial community. Such an infrastructure would facilitate the transfer of charge between dispersed mobile electron carriers (which would not have to move far), and act as a net to retain those carriers within the reach of the community that produced them. A key question is whether eDNA-protein interactions modulate this redox role? One possibility is that DNA-binding proteins are involved in regulating charge transfer, but this remains to be investigated in PA biofilms. However, we note that novel DNA-binding exoproteins have recently been identified in *Staphylococcus aureus* biofilms (Kavanaugh et al., 2019).

## 5 Experimentally accessing the interactome

Although cataloguing the extracellular compartment is now relatively straightforward, the next step–characterising the interactome of the molecules–may be more challenging. For example, although the identification of protein-protein interactions through approaches such as proximity labelling is now well-developed, the extensive processing of many secreted proteins (often at both N- and C-termini) complicates construction/secretion of the necessary chimeras. “Old school” chemical cross-linking remains an alternative.

Accessing the small molecule interactome requires rather more focused approaches, although there is evidence to suggest that these might be worthwhile. For example, the phenazines discussed above are known to be involved in a redox-based signalling pathway mediated by the intracellular protein, SoxR (Dietrich et al., 2006). But is SoxR the only protein that interacts with these pigments, or is the phenazine interactome much wider? This is potentially very open to exploration, especially given that certain phenazine derivatives have been shown to readily form radical-catalyzed S-conjugates with biogenic thiols, including proteins (Heine et al., 2016). It seems a short step from here to investigate the extracellular protein-phenazine interactome in PA.

Another relatively unaccessed small molecule interactome involves quorum sensing (QS); the colourful world of diffusible small “autoinducer” molecules that regulate the synthesis and export of a wealth of secreted factors (Chapon-Hervé et al., 1997; Pena et al., 2019). The conventional view is that they do so by binding to canonical transcriptional regulators (LasR, QscR, RhlR, PqsR) in the bacterial cytoplasm. These receptors subsequently undergo a conformational/oligomeric change to become active, thereby eliciting transcription specific target genes and operons (Seed et al., 1995; Whiteley and Greenberg, 2001). QS signals are abundant in the biofilm and potentially even accumulate in the extracellular matrix (Charlton et al., 2000). Crucially, several studies have hinted that QS signals also bind to other targets, although most of these studies have focused on identifying *intra*cellular binding partners (Baker et al., 2017; Dandela et al., 2018; Yashkin et al., 2021). This raises the question of whether QS molecules might bind to targets outside of the cell too. It turns out that they do.

The extent to which QS signals interact with extracellular proteins (and other molecules) is not well understood. It was found that the *pseudomonas* quinolone signal (PQS) interacts with outer membrane lipopolysaccharides. This stimulates MV generation and subsequent packaging of the QS molecule into those vesicles (Mashburn and Whiteley, 2005; Florez et al., 2020). Similarly, there is evidence that another QS autoinducer, *N*-(3-oxododecanoyl)-L-homoserine lactone, stimulates proinflammatory cytokine production in the cystic fibrosis airways, and other studies have demonstrated that widespread proteomic and respiratory perturbations are stimulated by this autoinducer in host cells (Mayer et al., 2011; Josephson et al., 2020). Recent technical advances in the field, such as thermal proteome profiling (Huber et al., 2015; Gaetani et al., 2019) may yet provide the means to expand our understanding of the QS interactome. However, in many respects, that’s the easy bit: the hard work will be in demonstrating the functional consequence(s) of such interactions.

## 6 Discussion

**FIGURE 2:**
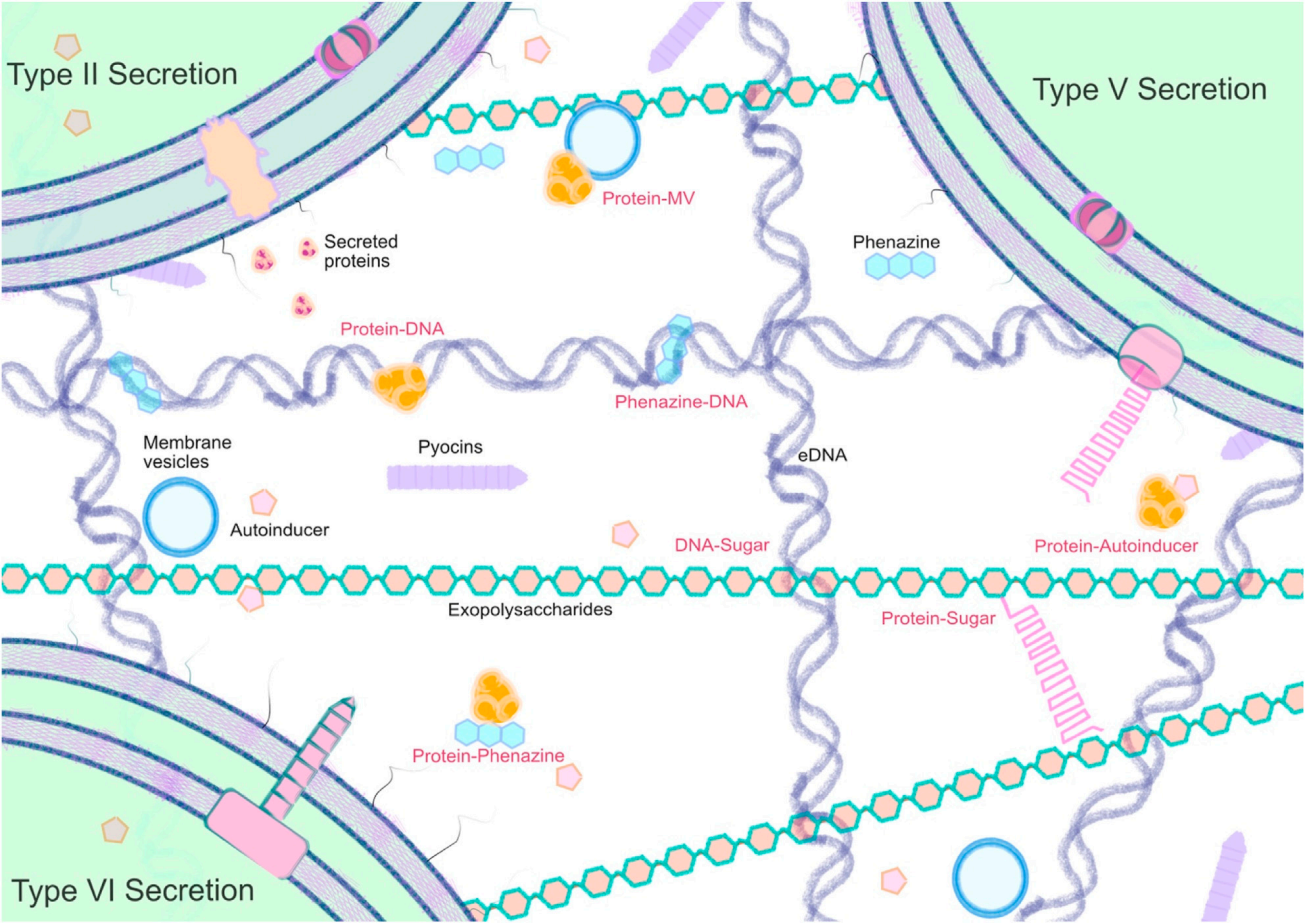
Simplified overview of the main classes of molecule present in the PA matrixome.

Through evolution, microbes have independently evolved diverse mechanisms for moving molecules from their cytosol to the extracellular space. In many cases, these exported molecules can help to shape the external environment, as well as being produced as a consequence of it. There is also increasing evidence to suggest that many secreted components—both small molecules and proteins—are selectively captured by the biofilm matrix, and interact with one another (Figure 2).

In this Perspectives piece, space limitations have necessarily forced us to focus on just a handful of specific examples. We note that most of these examples have been drawn from studies carried out in axenic cultures of defined *P. aeruginosa* strains. By contrast, in clinical scenarios, PA often shares its environment with a plethora of other organisms, including fungi, other bacteria (both Gram-negative and Gram-positive), phage, eukaryotic viruses, and protists—not to mention host cells—so the true extracellular interactome is likely to be far more complex than we currently appreciate. However, accessing this “new biology” is challenging; simply mixing microbial species together and hoping for the best does not capture the polymicrobial stability associated with many chronic infections (O’Brien and Welch, 2019; O’Brien et al., 2021).

In summary, we suggest that the biofilm matrix should be considered as a distinct extracellular compartment, and one which functions to capture and retain key secreted molecules. We note that these molecules may be secreted through well-characterized secretion systems, or may be delivered to the extracellular milieu via explosive cell lysis, or more sedately, through PQS-mediated vesiculation (Catalina et al., 2017). It is also likely that the biofilm matrix also acts to capture proteins derived from adjacent species, especially in mixed-species biofilms, or even from the host. This is an unexplored, and potentially very exciting area. Indeed, it has not escaped our attention that a better understanding of the binding determinants used by proteins to interact with specific biofilm matrix components could even offer a very effective route by which to target therapeutic agents to these structures. Now *that* would be a very useful outcome.

## Data Availability

The original contributions presented in the study are included in the article/Supplementary material, further inquiries can be directed to the corresponding author.
